# Gamified interactive e-books for bullying prevention: enhancing knowledge and motivation in Chinese primary schools

**DOI:** 10.3389/fpsyg.2025.1509549

**Published:** 2025-02-27

**Authors:** Jiawei Shao, Siti Nazleen Abdul Rabu, Chuang Chen

**Affiliations:** ^1^Centre for Instructional Technology and Multimedia, Universiti Sains Malaysia, Penang, Malaysia; ^2^School of Art and Design, Zhengzhou University of Industrial Technology, Zhengzhou, China

**Keywords:** gamification, gamified interactive e-books, bullying knowledge, bullying prevention, learning motivation, educational technology

## Abstract

Bullying is increasingly prevalent in Chinese schools, contributing to a rise in associated criminal cases. A key factor in this trend is the lack of comprehensive understanding of bullying. Studies indicate a clear correlation between the level of understanding of bullying and its frequency. The adverse effects of bullying are expected to persist into adulthood, particularly during the crucial phase of behavioral and cognitive development in elementary school, when children are most receptive to changes in behavior and attitudes. Traditional lecture-based methods used to teach bullying awareness to Chinese primary school students often result in boredom and disengagement, reducing their ability to comprehend and address bullying effectively. This study developed a gamified interactive e-book (GIEB) aimed at enhancing the motivation and anti-bullying knowledge of Chinese primary school students. A quasi-experimental design with pretest and posttest assessments was employed to evaluate the approach’s effectiveness. The study involved 60 third-grade students from a public primary school in Hefei, Anhui Province, China, who were randomly assigned to either an experimental group using the gamified interactive e-books (GIEB group) or a control group receiving traditional lectures (TL group). Findings indicated that the GIEB group showed significant improvements in motivation and understanding of bullying compared to the TL group. This research highlights the potential of gamified interactive e-books as effective educational tools for bullying prevention by making learning more engaging and effective. The practical implications of this study suggest that integrating gamified interactive e-books into the curriculum could significantly enhance students’ motivation to learn about bullying and their anti-bullying knowledge, ultimately contributing to more effective bullying prevention in schools.

## 1 Introduction

### 1.1 Definition, characteristics, and consequences of bullying

Bullying is a form of abuse, defined as aggressive behavior by an individual or a group that intentionally inflicts physical, verbal, or psychological harm on another person, repeatedly over time (Pan et al., 2017). Bullying typically occurs in interpersonal interactions and is characterized by an imbalance of power between the perpetrator and the victim (Olweus, 1993a). This power imbalance can manifest in various ways, such as differences in height, intelligence, popularity, or hierarchical positions between leaders and subordinates (Saibon, 2005; Aulia, 2016).

Bullying generally involves three key roles: the bully, the victim, and the bystander, all of whom contribute to reinforcing bullying behavior (O'connell et al., 1999). The consequences of bullying can be severe, affecting both the victim and the perpetrator. These effects include physical and psychological issues such as anxiety, depression, loneliness, and sleep disturbances, as well as academic difficulties (e.g., lower grades), health problems, and challenges in social adjustment (Pan et al., 2017; Mohamed El Swerky et al., 2022). More critically, these negative consequences can persist into adulthood.

Additionally, primary school children are in the early stages of behavioral and cognitive development, making them more receptive to corrective interventions (Fei et al., 2022). Therefore, implementing anti-bullying interventions during childhood is crucial to preventing long-term adverse effects on children’s development (Boden et al., 2016).

Numerous studies have shown that bullying is prevalent among children and adolescents and is becoming a global issue (Pan et al., 2017; Aulia, 2016; Fei et al., 2022; Song et al., 2019). The prevalence of bullying has been estimated to be between 15 and 25% in regions such as Europe, Australia, and the United States (Veenstra et al., 2005).

Due to the lack of sufficient attention given to the issue of bullying, its incidence continues to rise in Chinese schools (Pan et al., 2017; Fei et al., 2022; Wong et al., 2008). A national survey in China found that 57.29% of junior high school students experienced school bullying at least once in the previous year (Song et al., 2019). Furthermore, Chinese prosecutors prosecuted over 260 individuals for serious school bullying incidents between January and September 2023 (China Internet Information Centre (CIIC), 2024).

Between January and April 2024, three junior high school students in Hebei Province, who had persistently bullied their classmates, brutally murdered their victims. Additionally, a primary school student in Changsha, Hubei Province, took their own life due to prolonged bullying by classmates. In Henan Province, long-term bullying led to an incident where the victim’s parents resorted to violence against the perpetrator.

With the widespread adoption of information technology and the internet, school bullying in China has become increasingly diverse. What initially involved verbal insults and physical assaults has now expanded to include psychological bullying and cyberbullying.

Cyberbullying is defined as bullying conducted through the internet or digital devices, characterized by an imbalance of power, repetitive behavior, and the intent to harm the victim. Its anonymity and rapid dissemination make it particularly threatening to victims (Olweus, 1993b; Langos, 2012; Kowalski and Limber, 2007; Terry and Cain, 2016; Marinoni et al., 2024). This distinction positions cyberbullying as an emerging and increasingly concerning form of school bullying.

### 1.2 Prevalence and challenges of bullying in China

Unlike traditional bullying, cyberbullying transcends temporal and spatial boundaries, potentially subjecting victims to prolonged psychological stress. The prevalence and severity of this form of bullying have significantly increased.

Research indicates that the victimization rate of traditional school bullying in China is as high as 66%, with a perpetration rate of 68%. Meanwhile, the victimization rate of cyberbullying reaches 72%, with a perpetration rate of 60%. Since many of these findings rely on self-reported data, the actual figures may be underestimated (Chan and Wong, 2015).

These statistics highlight the widespread nature of school bullying and underscore the urgent need for effective intervention strategies.

To address this issue, Chinese researchers have conducted various studies. For instance, Du and Li (2019) implemented a program aimed at fostering students’ social–emotional skills to prevent school bullying, which led to a reduction in bullying incidents. Similarly, Pan (2019) developed and executed a peer protection intervention program in a middle school, resulting in an increased willingness and ability among students to protect their peers, as well as a decrease in the number of bullying victims.

These studies demonstrate that enhancing students’ social skills and fostering peer support are effective strategies for bullying prevention. However, research on school bullying prevention and intervention in China remains limited. Most studies are still in the design phase, and empirical research is relatively scarce.

Lack of knowledge about bullying behavior is one of the key reasons for its increasing prevalence. Saibon et al. (2012) asserted that contemporary youngsters possess insufficient understanding of bullying behavior and that there is a direct relationship between the extent of knowledge regarding bullying and the occurrence of bullying incidents. This suggests that the less students know about bullying behavior, the more likely they are to become either perpetrators or victims (Saibon et al., 2017).

Furthermore, insufficient knowledge about bullying can lead to students’ misperceptions of the phenomenon. They may unknowingly engage in behaviors that constitute bullying or fail to recognize when they themselves are being bullied (Saibon, 2009). Chu et al. (2019) emphasized that increasing students’ understanding of bullying is essential for fostering positive changes in their attitudes and behaviors. However, primary school students generally lack awareness of bullying (Osiesi et al., 2023; Widayati et al., 2021; Manik and Sinaga, 2022). The Chinese Ministry of Education explicitly highlights the need to raise students’ awareness of bullying in its *Prevention of Bullying among Primary and Secondary School Students Work Programme of Special Control Actions* (Ministry of Education of the Peoples Republic of China, 2021; Osiesi et al., 2023).

Schools in most countries, including China, continue to rely on traditional methods to raise students’ knowledge and awareness of bullying. These methods include, but are not limited to, group discussions, lecture seminars, and large-scale school assemblies. However, such approaches often yield limited effectiveness (Widayati et al., 2021; Beale and Hall, 2007; Diamanduros et al., 2008; Keith and Martin, 2005).

Some scholars have highlighted the difficulties students face in enhancing their understanding of bullying behavior through traditional methods, particularly those in primary education. Young students, especially those newly entering primary school, often lack intrinsic motivation to learn about bullying. Traditional approaches struggle to create an engaging learning environment, leading to boredom and disengagement, which ultimately hinders students’ ability and willingness to further explore bullying-related knowledge (Saibon et al., 2018; Wahab et al., 2015).

Despite recent efforts to incorporate innovative teaching methods to improve students’ knowledge of bullying, traditional lecture-based instruction remains the predominant approach in many Chinese schools (Xiong, 2019; Liu, 2023).

### 1.3 Digital and gamified learning: a promising solution

With advancements in digital technologies, e-books have gradually emerged as a digital alternative to traditional paper books, providing users with a convenient reading experience through electronic devices (Kong et al., 2018). Compared to traditional paper books, interactive e-books integrate multimedia and interactive features, enhancing not only students’ reading experience but also their learning outcomes by increasing motivation, engagement, and satisfaction (Bus et al., 2019). Therefore, incorporating interactive e-books into learning activities can better utilize digital features, offering innovative support and exploratory opportunities for students (Geiger et al., 2010; Muranov et al., 2023).

Gamification was initially defined as “the use of game design elements in a non-game environment” (Deterding et al., 2011). Subsequent studies have expanded on this definition. Huotari and Hamari (2012) emphasized that its core lies in implementing motivational strategies to influence user behavior, while Hamari et al. (2019) described gamification from a broader perspective as a cultural and societal phenomenon in which reality becomes increasingly game-like, fostering skill development, motivational benefits, and engagement. This suggests that gamification is not merely a technical tool but an innovative strategy designed to enhance experiences and motivational outcomes.

As an educational technique, gamification has garnered significant attention in both practical applications and academic research. It has been implemented across various fields to varying degrees, with education being a primary focus (Zhao et al., 2021; Mehta et al., 2022; Chen et al., 2023). Integrating game elements into real or virtual learning environments can create a more engaging and enjoyable experience for students. By incorporating these gamified components into meaningful educational content, teachers can provide students with a more immersive and interactive learning process (Subhash and Cudney, 2018). Research has shown that this approach effectively enhances student engagement, fosters deeper learning, and cultivates a more positive attitude toward education (Hassan et al., 2021). Furthermore, gamified learning, which integrates game elements into traditional classroom instruction, has been found to significantly improve students’ motivation, learning abilities, participation, and social interaction (Zainuddin et al., 2020; Baxter et al., 2015).

### 1.4 Current study

The emergence of gamification presents new opportunities for enhancing Chinese primary school students’ knowledge of bullying behavior. Integrating gamification with interactive e-books has the potential to improve students’ motivation and understanding of bullying. Therefore, this study aimed to design and develop a gamified interactive e-book and evaluate its impact on enhancing students’ knowledge of bullying by increasing their motivation. Based on this objective, we formulated the following research questions.

1. To what extent do gamified interactive e-books enhance primary school students’ knowledge of bullying compared to traditional lectures?
2. To what extent do gamified interactive e-books lead to greater learning motivation in primary school students compared to traditional lectures?

## 2 Literature review

### 2.1 Gamification

Integrating gamification with educational content and knowledge has emerged as a highly engaging and motivating educational strategy in the era of interactive media and the widespread popularity of games (McGonigal, 2012; Majuri et al., 2018; Sailer and Homner, 2020). Gamification is generally defined as the incorporation of game elements and mechanics into non-game contexts, with the primary objective of enhancing user engagement to improve activity levels and overall performance (Deterding et al., 2011; Suh et al., 2016; Bizzi, 2023). As a result, gamification strategies have been widely adopted in education. Studies have shown that gamification significantly enhances learning motivation, engagement, and outcomes (Pedreira et al., 2015; Zhao et al., 2021; Chen et al., 2023).

Werbach et al. (2012) identified dynamics, mechanics, and components as the three core elements of gamification design. *Dynamics* encompass a sense of achievement, social interaction, exploration and discovery, competition, and cooperation, aiming to shape the overall user experience. *Mechanics* include tasks and goals, reward systems, leaderboards and competition, feedback mechanisms, and other strategies that serve as essential tools to realize dynamics. *Components*, such as role-playing, progress tracking, and virtual economies, represent specific implementations designed to trigger user behaviors. Depending on the specific application context and learning objectives, these game elements can be flexibly combined to effectively enhance user engagement, address real-world challenges, achieve learning goals, and ultimately deliver the desired learning outcomes (Zainuddin et al., 2020; Werbach et al., 2012).

There is a growing body of data suggesting that gamification is increasingly recognized as an effective supporting technique in learning. In recent years, traditional learning methods have gradually lost their appeal to learners (Fortus and Vedder-Weiss, 2014). Gamification has emerged as a promising solution to this issue. As an innovative educational strategy, gamification integrates game elements into non-game contexts to enhance user engagement, learning motivation, and behavioral change (Deterding et al., 2011).

Research has shown that gamification can significantly improve students’ academic performance. For instance, Puig et al. (2022) integrated gamification into geometry learning content, significantly boosting students’ motivation and performance while helping them understand the definitions and features of geometric shapes more intuitively. The study also emphasized the importance of contextualized learning and dynamic difficulty adjustment and suggested integrating digital technologies to further enhance learning outcomes.

Beyond academic performance, gamification has also proven effective in intervening in attitudes and behaviors. For example, Saleme et al. (2019) applied gamification technologies to a social marketing program, significantly improving children’s affective empathy and empathetic behavior. Similarly, Alsaleh and Alnanih (2020) developed a health-focused gamified application that improved diabetic children’s knowledge of healthy eating and effectively altered their dietary habits.

Despite its potential in education and behavioral interventions, the application of gamification in bullying prevention remains limited. Álvarez-Bermejo et al. (2016) developed a gamified application aimed at preventing bullying behavior triggered by racial stigma. The study showed that the application helped teachers better understand student interactions and reduced bullying by adjusting group compositions. However, challenges such as high costs and resource allocation have hindered its practical implementation.

Gamification also shows growing potential in addressing sensitive topics. For example, Ros Morente et al. (2018) examined the effects of gamified software, such as Happy 8–12 and Happy 12–16, on students’ emotional competencies. Their findings demonstrated that these tools significantly enhanced students’ emotional management skills, reduced anxiety, and improved academic performance, highlighting the critical role of emotional competencies in improving students’ wellbeing and learning outcomes.

In the area of gender-based violence (GBV) prevention, a gamified platform has demonstrated positive results (Gini et al., 2024). By comparing two versions of the platform (individual and cooperative), the study found that the platform effectively promoted awareness of healthy relationships and social interaction. Although sensitive topics may elicit negative emotions such as anger and frustration, the platform also fostered user engagement and interest.

Moreover, the integration of virtual reality (VR) technology and the metaverse offers new possibilities for bullying prevention. For example, Sánchez-Romero and Muñoz-Jiménez (2024) proposed the development of 3D educational content in the metaverse to address “proximity cyberbullying.” This gamified approach combines immersive interaction designs to provide effective education and training tools for students and teachers. Another study found that VR interventions significantly enhanced empathy, reduced traditional bullying behaviors, and improved school belonging and bystander intervention willingness, though their impact on cyberbullying remained limited (Ingram et al., 2018).

Further supporting the potential of digital tools in bullying prevention, the *FearNot!* project employed a Virtual Learning Environment (VLE) to simulate bullying scenarios and promote empathy and defender behavior among students aged 7–11 (Vannini et al., 2011). The study involved 1,186 students from the UK and Germany, with results showing a significant increase in cognitive empathy and defender behavior among German students, whereas no significant effects were observed in the UK sample, possibly due to their higher baseline awareness of bullying. The study highlights the value of digital tools in bullying prevention and emphasizes the influence of cultural and educational contexts on intervention outcomes.

Moukram et al. (2023) conducted a comprehensive review of the use of game-based resources, including game-based learning, serious games, and gamification, in bullying prevention. They found that current research primarily focuses on serious games and game-based learning, with limited empirical studies on gamification. Similarly, Nurtanto et al. (2021) highlighted through a systematic literature review that gamification positively impacts learning across emotional, cognitive, behavioral, and performance dimensions. However, further research is needed to explore its application in specific contexts.

While numerous empirical studies have demonstrated the efficacy of gamification in various educational contexts, its limitations have also been widely acknowledged. For example, Widodo and Rahayu (2019) employed an offline gamified learning tool to enhance students’ mathematical performance, yet the findings revealed no significant improvement in mathematical proficiency. Similarly, Choi et al. (2022) developed a gamified learning tool to improve students’ foundational knowledge of blockchain, but the study found no substantial enhancement in their comprehension of software and hardware or digital literacy. These studies suggest that gamified learning tools with simple designs—relying on limited elements such as points and competition mechanisms—may not yield significant educational benefits, particularly when implemented offline and without integration with advanced digital or mobile technologies.

Kyewski and Krämer (2018) further argued that the use of basic game components, such as points and leaderboards, is insufficient to enhance student motivation and learning outcomes. Instead, incorporating more sophisticated pedagogical strategies alongside digital tools can better engage digitally native learners and foster inquiry-based learning (Geiger et al., 2010). Moreover, Faure-Carvallo et al. (2022) highlighted the importance of social elements in gamification, asserting that most current designs primarily focus on individual behaviors. Effective gamification, they argued, requires parallel support from teachers, instructional texts, and peers. Additionally, competitive mechanisms in gamification demand timely feedback to sustain student engagement throughout the learning process.

Concerns about gamification in school environments have also been raised. For instance, overuse of digital gamification tools may lead to adverse health effects, such as vision impairment in students (Faure-Carvallo et al., 2022). Furthermore, gamification strategies cannot entirely replace traditional teaching methods. Studies have shown that traditional approaches remain essential for knowledge acquisition, as they may slow down the cognitive process, allowing students to analyze and assimilate information more effectively (Zhukova et al., 2023; Hung, 2018).

The literature indicates that while empirical studies combining gamification with bullying prevention remain relatively scarce (Álvarez-Bermejo et al., 2016; Moukram et al., 2023), existing research has demonstrated the potential of gamification in this field (Ros Morente et al., 2018; Gini et al., 2024; Ingram et al., 2018; Sánchez-Romero and Muñoz-Jiménez, 2024). However, most of these studies have been conducted in Western contexts, with no relevant empirical research identified in China. This geographical gap provides an important opportunity for this study to make a unique contribution to the field.

Existing research indicates that integrating gamification with digital technologies can significantly enhance students’ learning motivation and performance (Geiger et al., 2010; Puig et al., 2022), while also demonstrating positive effects in emotional management and education on sensitive topics (Ros Morente et al., 2018; Gini et al., 2024). Digital tools have also shown substantial potential in bullying prevention (Vannini et al., 2011). Furthermore, emerging technologies such as virtual reality (VR) and the metaverse offer innovative approaches to addressing bullying (Ingram et al., 2018; Sánchez-Romero and Muñoz-Jiménez, 2024). However, some studies suggest that the mere adoption of simple gamification elements, such as points and leaderboards, is insufficient to enhance students’ motivation and learning performance (Kyewski and Krämer, 2018). To create an engaging and motivating system, it is essential to incorporate design elements aligned with the specific purposes and context, such as socialization and timely feedback (Pedreira et al., 2015; Kyewski and Krämer, 2018; Koivisto and Hamari, 2019; Bassanelli et al., 2024; Fortes Tondello et al., 2017).

Based on these findings, this study aims to develop an efficient digital gamification approach by integrating dynamic adjustment, immersive interaction, and socialization design. The goal is to enhance Chinese primary school students’ understanding of bullying and provide innovative tools for bullying prevention.

### 2.2 Knowledge on bullying behavior

Bullying is the act of an individual or group repeatedly and deliberately causing harm or posing a threat to another person or group (Rigby, 2000). Bullying behavior is currently prevalent among students and has evolved into various forms over time. The main types of bullying include physical bullying, verbal bullying, social bullying, and cyberbullying. Some of these behaviors are overt and easily identifiable, such as physical and verbal bullying, while others, like social bullying and cyberbullying, are more subtle and harder to detect.

Physical bullying encompasses actions such as hitting, kicking, shoving, tripping, and damaging property. Verbal bullying includes behaviors such as using derogatory language, mocking, making racist comments, instilling fear, and verbal abuse. Covert bullying, also known as social bullying, can occur without the victim’s physical presence, making it difficult to identify. Its primary aim is to damage the victim’s social reputation or humiliate them (Saibon et al., 2017). Examples of social bullying include spreading rumors, exclusion, and coercion.

Cyberbullying can be both overt and covert, involving the use of the internet and other ICT devices. It includes sending harmful images or messages (Willard, 2005), such as text messages, emails, social media harassment, and blackmail.

Students who experience bullying may endure physical or psychological harm, including conditions such as sadness, anxiety, hyperactivity, loneliness, sleep disturbances, physical ailments, loss of appetite, and nausea (Ford et al., 2017; Savahl et al., 2019; Wolke et al., 2001). Furthermore, bullying has been linked to an increased risk of suicidal thoughts among students (Willard, 2005).

Most bullying prevention programs primarily focus on educating students or teachers on how to take action to stop bullying. However, these interventions often occur after the bullying behavior has already taken place, by which point the student may have already suffered physical and mental harm (Saibon et al., 2017).

A key contributing factor to bullying behavior is students’ insufficient awareness and understanding of bullying. Research has demonstrated a significant inverse relationship between students’ knowledge of bullying and their likelihood of engaging in bullying behavior (Yuniliza, 2020; Yani, 2017). Consequently, an increasing number of studies have sought to prevent bullying by enhancing students’ knowledge of bullying behavior. However, traditional methods appear to be less effective in achieving this goal (Widayati et al., 2021), primarily because students often find these methods unengaging and lack motivation to learn through them (Saibon et al., 2018; Wahab et al., 2015).

A growing number of researchers have sought to increase student motivation by creating engaging learning environments to enhance students’ knowledge of bullying.

Beran and Shapiro (2005) evaluated the effectiveness of the *Project Ploughshares Puppets for Peace* initiative in Canadian elementary schools. This program utilized puppet performances to reenact scenarios depicting the occurrence and resolution of bullying, aiming to enhance students’ understanding of bullying behavior. However, the findings revealed no significant improvement in students’ comprehension of bullying.

Saibon et al. (2017) employed creative teaching methods to improve Malaysian secondary school students’ knowledge of bullying behavior. The results demonstrated a significant improvement in students’ understanding of bullying behavior following the intervention. In their study, Saibon et al. (2017) emphasized the critical role of motivation in enhancing students’ comprehension of bullying. During study interviews, students described previous traditional methods as boring, which led to disengagement and a diminished willingness to continue learning.

Donohoe and O'Sullivan (2015) enhanced Irish primary school students’ knowledge of bullying through role-playing and discussion, leading to a reduction in bullying incidents. Their findings demonstrated a substantial improvement in students’ understanding of bullying behavior, resulting in a notable decrease in bullying occurrences within schools.

Rončević Zubković et al. (2022) examined whether digital games designed for bullying prevention enhanced knowledge and empathy toward bullying among European secondary school students. They also explored variations in students’ gaming experiences concerning their knowledge of bullying and empathy toward victims. The findings demonstrated a significant increase in students’ understanding of bullying and empathy for victims.

The literature review revealed that research on enhancing students’ knowledge of bullying behavior through the development of anti-bullying programs remains scarce. Empirical studies that aim to enhance students’ motivation and, ultimately, their knowledge of bullying behavior by creating enjoyable learning environments are even rarer. Several key issues were identified:

1. The majority of studies are concentrated in European countries, Canada, and Malaysia, with only a limited number conducted in China.
2. Most studies focus on creating engaging offline learning environments, while there is a lack of research integrating digital technology. Empirical studies on gamification in this field have not been identified.

Therefore, this study selected a Chinese school safety education curriculum on anti-bullying as the foundation for content design and developed a gamified interactive e-book (hereafter referred to as GIEB) to enhance Chinese primary school students’ motivation and knowledge about bullying.

## 3 The GIEB design process

### 3.1 System architecture of GIEB

**Figure 1:**
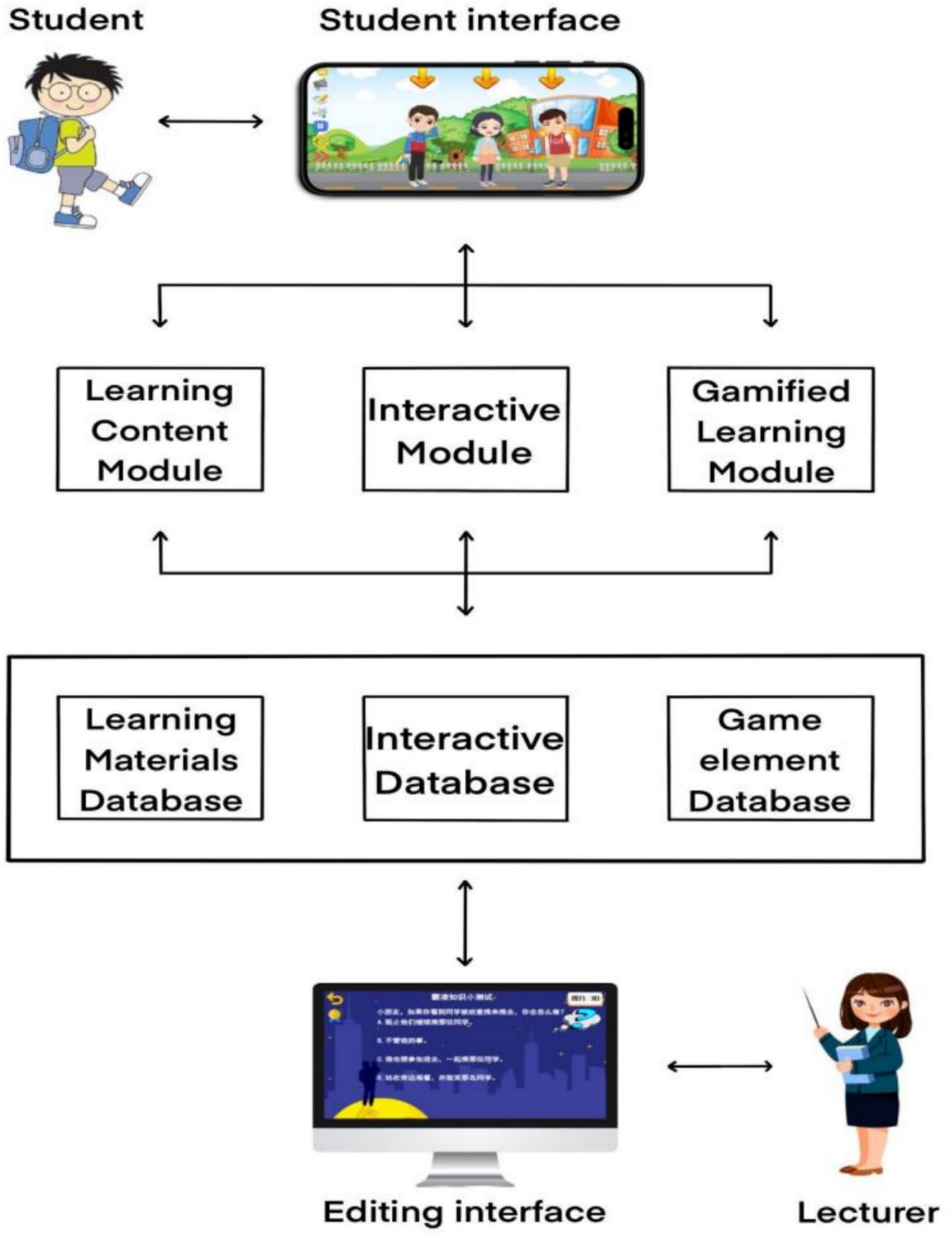
System architecture of GIEB.

In this study, the Yoya interactive e-book development program, created by Xiamen Yoya Network Technology Company, was used to develop an interactive e-book aimed at enhancing awareness of bullying among Chinese primary school students. Yoya offers a wide range of character designs, scene elements, and intelligently matched smooth voiceovers. It also includes common animation templates, image text, and background sound effects. Additionally, Yoya enables designers to create assessments and engage in interactive teaching with students. However, there are still limitations regarding product stability and user experience. This e-book consists of three primary modules: the learning content module, the interactive module, and the gamified learning module, as illustrated in Figure 1.

The Gamified Interactive E-book (GIEB) employs a three-module system architecture to enhance its educational effectiveness. The Learning Content Module serves as the foundation, presenting anti-bullying curriculum content adapted from the Chinese school safety education curriculum. This module utilizes a contextual story to engage students and facilitate knowledge acquisition. Built using the Yoya platform, the Interactive Module offers a series of tasks and challenges directly related to the bullying scenarios introduced in the story. These interactive elements include quizzes, decision points requiring user input to influence the narrative flow, drag-and-drop activities, and other engaging exercises that promote active learning. Lastly, the Gamified Learning Module, also facilitated by Yoya, incorporates game mechanics such as points, leaderboards, and badges to further motivate student engagement and reinforce learning. This modular structure, combined with Yoya’s versatile features, provides a comprehensive and engaging learning experience aimed at improving both understanding and motivation related to anti-bullying behavior.

### 3.2 Theoretical framework of the GIEB

The study grounds the GIEB’s design in Self-Determination Theory (SDT), suggesting that the interactive elements and gamified features were strategically implemented to foster students’ autonomy, competence, and relatedness, thereby enhancing motivation and learning (Vallerand, 2000; Kam and Umar, 2018). Self-Determination Theory (SDT) is a motivational framework that identifies three fundamental psychological needs: autonomy, competence, and relatedness. Satisfying these needs fosters intrinsic motivation, which is a critical factor in cognitive, social, and physical development (Vallerand, 2000). Intrinsically motivated individuals are more inclined to engage in learning activities that align with their interests and preferences, thus enhancing knowledge and skills in a personally meaningful way (Ryan and Deci, 2000; Donohoe and O'Sullivan, 2015). Research has consistently shown a positive correlation between intrinsic motivation and persistence in learning, indicating that students with higher intrinsic motivation are more likely to sustain their engagement in educational activities (Vallerand and Bissonnette, 1992; Rončević Zubković et al., 2022).

Building on these findings, Sailer and Homner (2020) argued that gamification can effectively enhance students’ motivation if it satisfies all three SDT needs. However, addressing these needs uniformly across diverse learners presents challenges, as individuals may prioritize different psychological needs based on their personal preferences and motivations. Research highlights that demographic factors, particularly gender differences, influence students’ engagement with gamified systems (Zahedi et al., 2021).

Zahedi et al. (2021) conducted a mixed-methods study on the role of gender in gamification’s impact on computer science students’ academic performance and identity development. Their findings indicate that gamification’s effectiveness varies among learners, with gender-based differences in engagement and performance suggesting the need for more personalized gamification strategies. These results align with the SDT framework, emphasizing that learners prioritize different psychological needs, necessitating tailored gamification approaches. Consequently, frameworks such as the Hexad Player Types, BrainHex, and the Big Five personality model have been developed to analyze player types and optimize gamification strategies (Pessoa et al., 2023; Vera Cruz et al., 2023; Alalgawi and Sadkhan, 2022). These models provide insights into designing gamified systems that align with diverse learner characteristics, ensuring a more inclusive and effective implementation.

**Figure 2:**
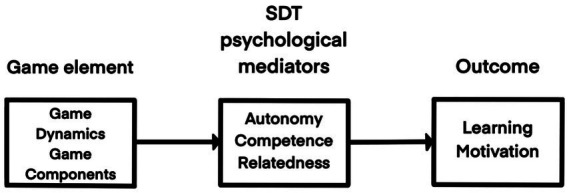
SDT-based design framework for gamification.

In their study, Kam and Umar (2018) proposed a gamification design framework based on self-determination theory. This framework, illustrated in Figure 2, aims to enhance students’ intrinsic motivation by deliberately incorporating game dynamics and components to fulfill three psychological needs. Independent decision-making is essential for students to develop a sense of autonomy. Educational settings should implement gamification while respecting students’ freedom of choice, providing multiple attempts, reducing the risk of task failure (e.g., failure does not result in a loss of in-game points), and allowing students to choose their preferred learning mode or style.

A sense of competence is fostered by providing students with tasks or challenges that best match their abilities and offering positive, timely feedback upon task completion, reinforcing their sense of achievement. A sense of relatedness reflects interconnection and social value. For instance, students can engage in discussions during the learning process and showcase their achievements on the profile screen, fostering a collaborative and engaging learning environment.

### 3.3 The GIEB design process

**Figure 3:**
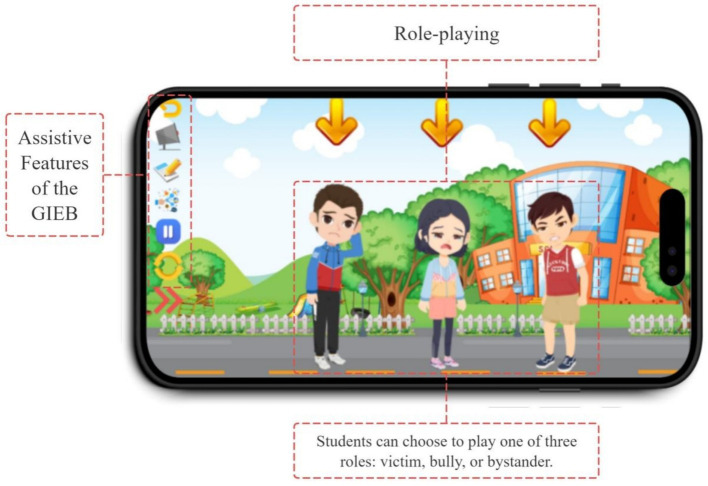
Role play selection screen.

The GIEB (Gamified Interactive E-book) is designed to provide students with an immersive and engaging learning experience about bullying. Students can choose to play one of three roles: victim, bully, or bystander (see Figure 3). This role-playing aspect, facilitated by the Yoya development platform, allows for branching narratives or simulations where student choices directly impact the story’s progression.

**Figure 4:**
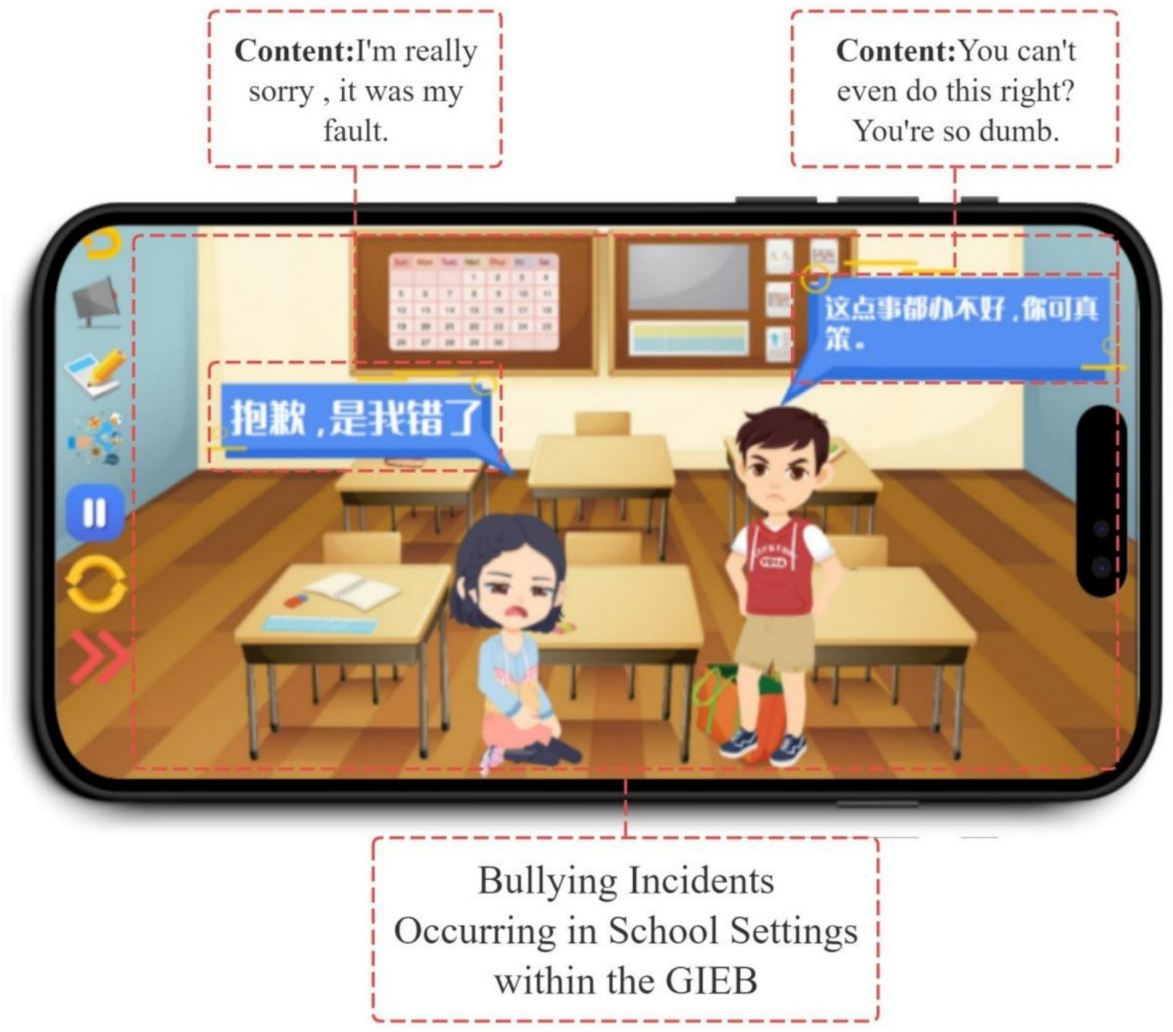
Bullying behavior in school scenes.

**Figure 5:**
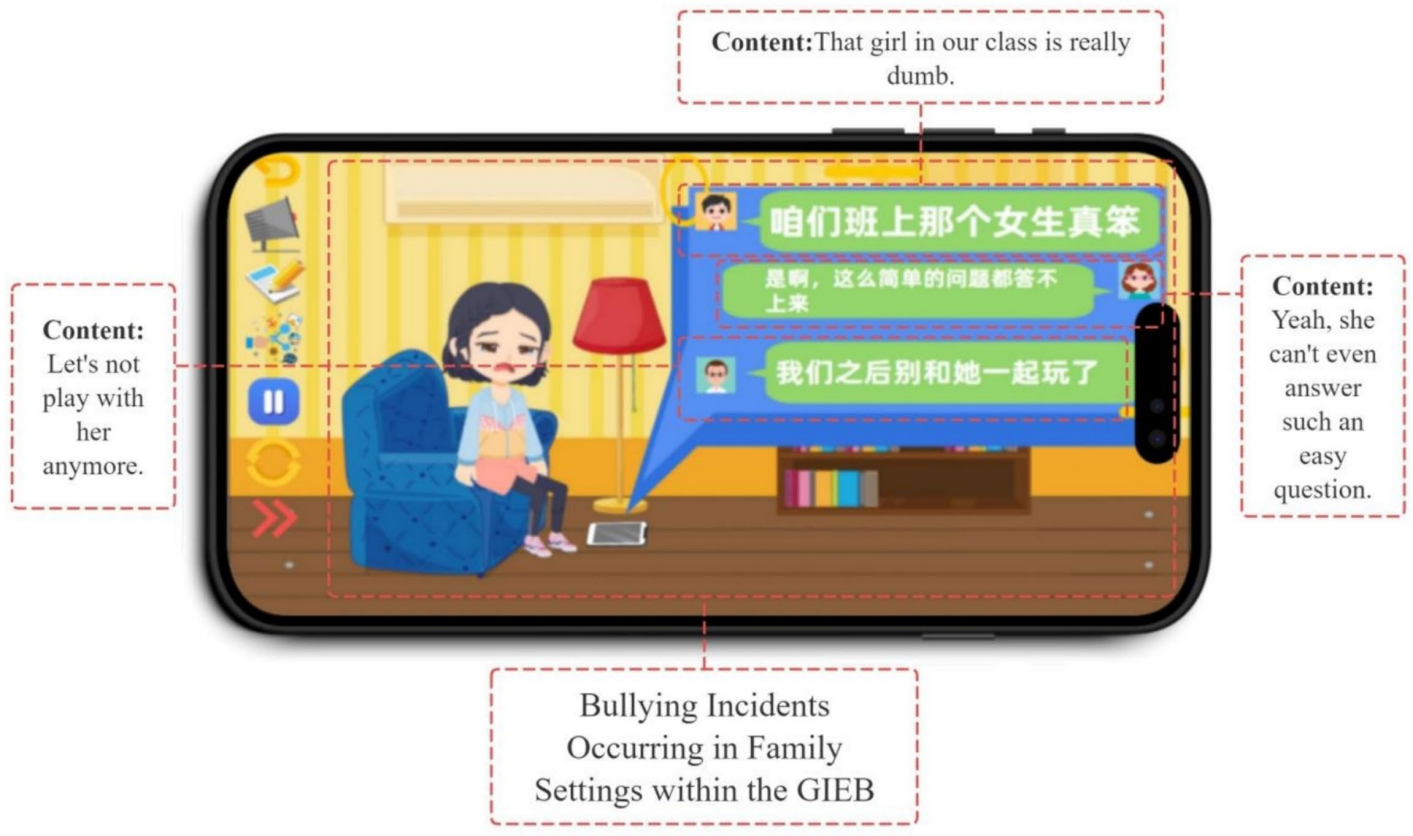
Cyberbullying in a family scenario.

Bullying scenarios within the GIEB occur in various settings, including school (see Figure 4), home, and online through social media (see Figure 5). This variety of scenarios, coupled with the need for students to make decisions within those contexts, highlights Yoya’s capacity to support interactive storytelling and decision-making simulations.

**Figure 6:**
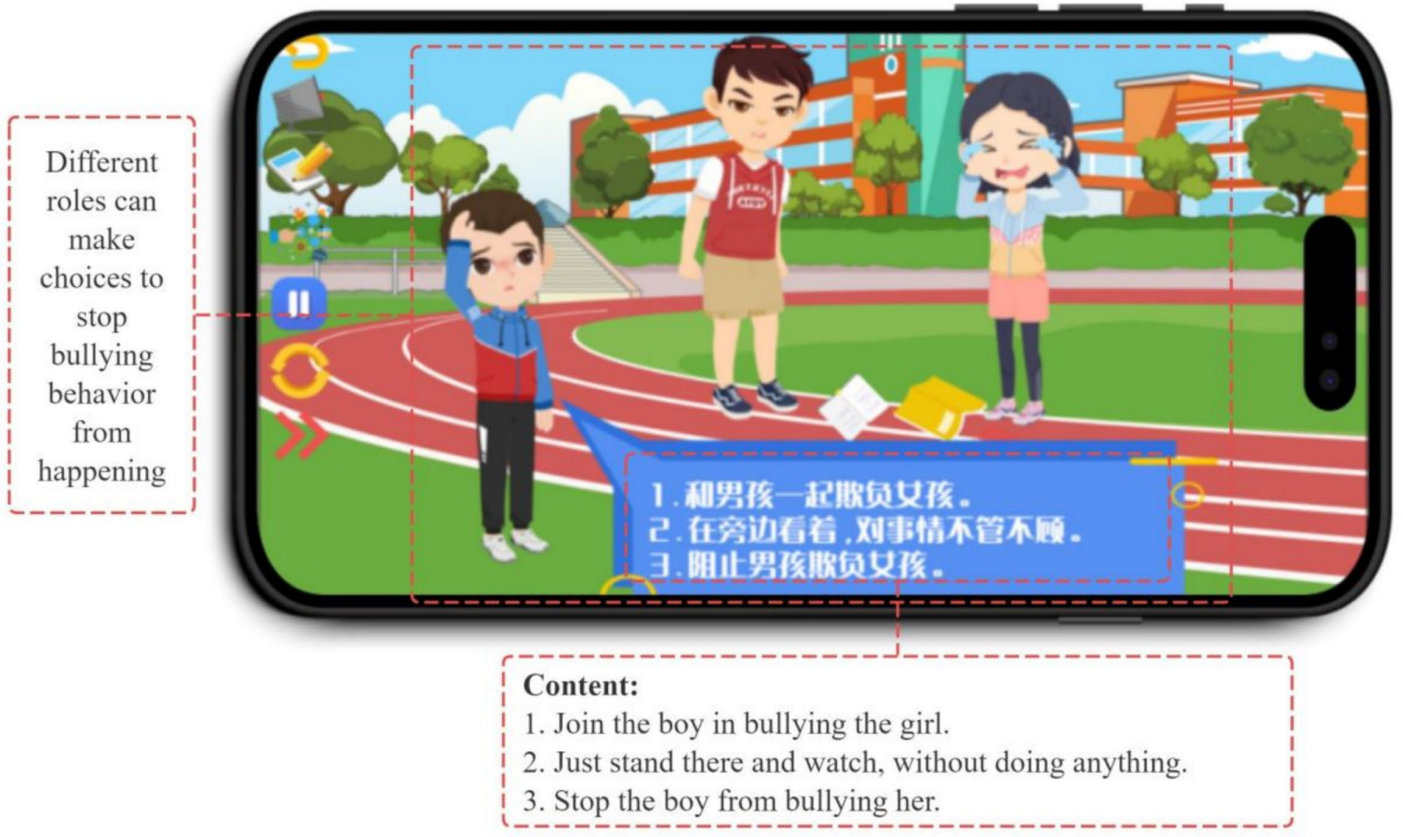
Different roles can make choices to stop bullying behavior from happening.

By taking on different roles, students gain a multifaceted understanding of bullying. For instance, playing the victim teaches them how to protect themselves, while playing the bully illuminates the negative consequences of such actions and the positive outcomes of choosing kindness. The bystander role allows students to recognize bullying behaviors and learn effective intervention strategies (see Figure 6).

Throughout the GIEB experience, students receive timely feedback on their choices and actions. Inappropriate behavior is immediately addressed, offering guidance on appropriate responses to similar situations and clarifying whether the observed action constitutes bullying. Yoya’s ability to integrate such feedback mechanisms strengthens the learning process.

**Figure 7:**
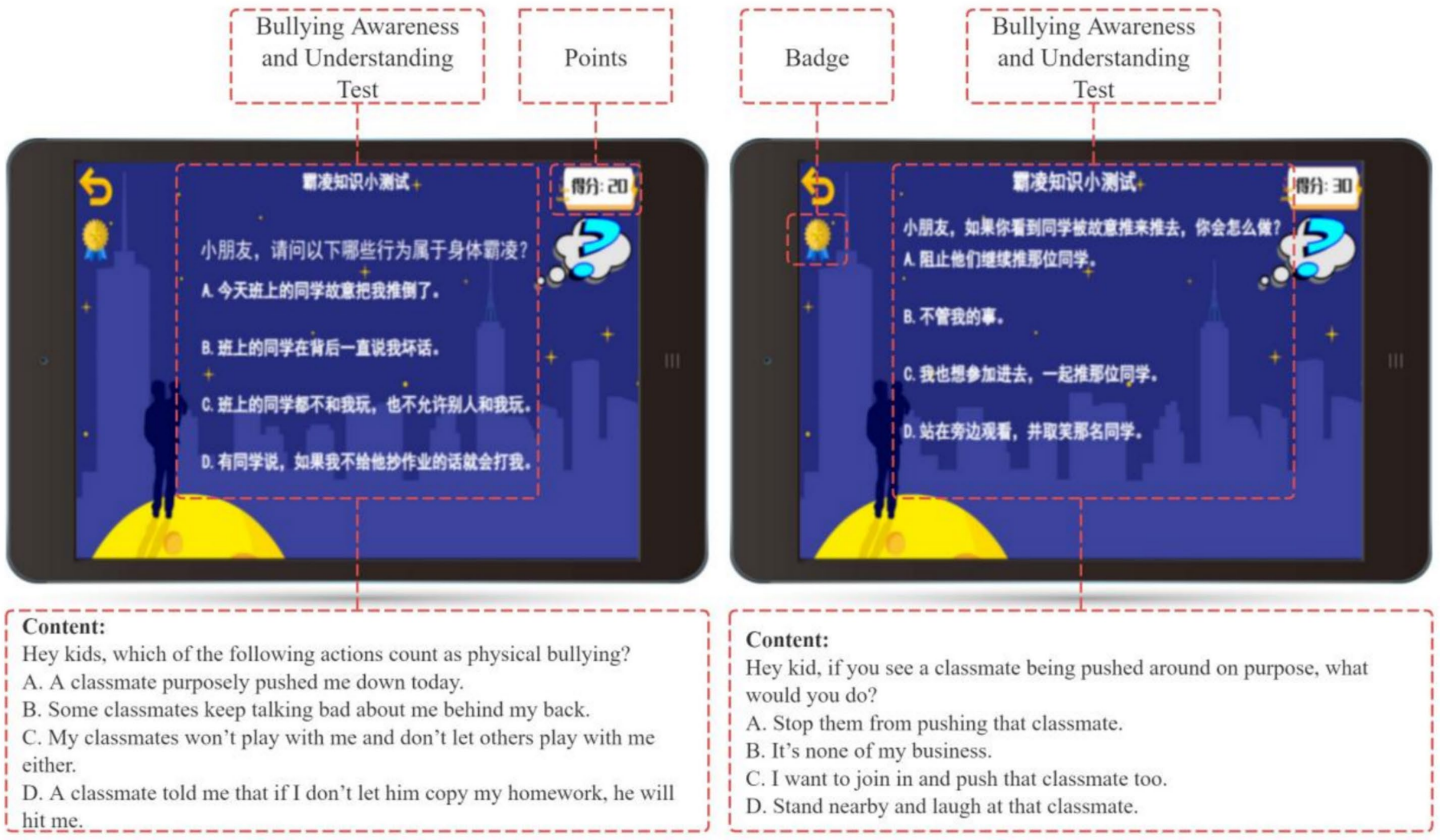
Bullying awareness and understanding test.

**Figure 8:**
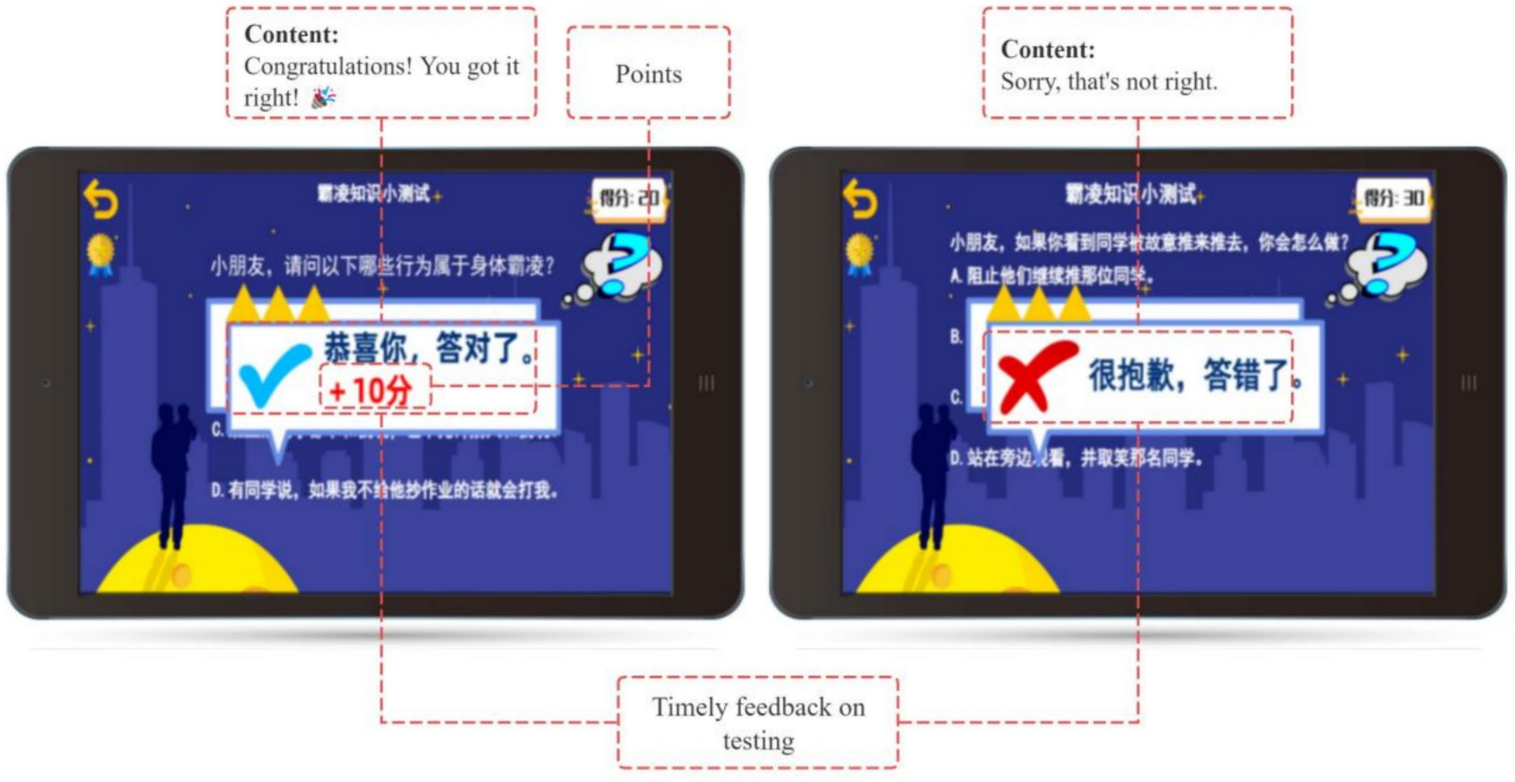
Timely feedback on testing.

After each story segment, students complete a repeatable quiz to reinforce their knowledge and assess their ability to apply the learned concepts (see Figure 7). Correct answers earn points, which can be redeemed for badges, adding a gamified element to the learning process. Yoya’s support for these gamified features enhances student motivation and fosters a sense of competence (see Figure 8).

Furthermore, the GIEB encourages social interaction by allowing students to share their points and badges with peers. This feature not only promotes a sense of relatedness among students but also highlights Yoya’s capacity to integrate social elements like leaderboards and sharing functionalities within the e-book. Ultimately, the GIEB’s combination of role-playing, interactive scenarios, and gamified elements creates a dynamic learning environment that promotes a deeper understanding of bullying and encourages positive social interaction.

Table 1 summarizes the key functions of the GIEB and how they were implemented using the Yoya platform.

**Table 1:** Key functions of the GIEB.

Function	Description	Yoya’s role
Role-playing	Students can experience bullying scenarios from different perspectives (victim, bully, bystander).	Enables branching narratives and simulations where user choices influence the story.
Scenarios & decision-making	The GIEB presents diverse bullying scenarios in various settings, requiring students to make choices that impact the outcomes.	Supports the development of interactive storytelling and decision-making simulation.
Feedback & rewards	The system provides immediate feedback on students choices and rewards correct answers with points and badges.	Facilitates the integration of feedback mechanisms and gamified elements to enhance learning and motivation.
Social features	Students can share their points and badges with peers.	Offers features to incorporate social elements, such as leaderboards and sharing functionalities, to promote interaction and a sense of community.

### 3.4 Game elements in GIEB

Informed by SDT, this study adopts the gamification design framework proposed by Kam and Umar (2018), integrating carefully selected game elements to support students’ motivation. These elements were implemented to foster a learning environment that aligns with SDT principles, thereby promoting student engagement and improving learning outcomes.

In terms of autonomy, students are empowered to make independent choices throughout the GIEB experience. They freely select from three distinct roles—victim, bully, or bystander—and have full control over the interactive e-book’s operation. They can start, pause, repeat, or skip sections of the story at any time, allowing them to learn at their own pace and in a style that suits them best. This flexibility aligns with Buckley and Doyle’s (2017) identification of diverse gamification elements and ensures that the learning experience caters to individual preferences.

In terms of competence, the GIEB provides students with a sense of accomplishment and progress. Questions embedded within the school and home-based storylines challenge students to think critically and apply their knowledge. Timely feedback is given after each choice or quiz answer, reinforcing correct responses and addressing incorrect ones with explanations. Earning points for correct quiz answers and redeeming those points for badges further contributes to a feeling of competence and progress.

Finally, in terms of relatedness, social interaction is encouraged through features that allow students to share their earned points and badges with their peers. This fosters a sense of community and provides opportunities for discussion and collaboration, further enhancing student engagement and knowledge sharing.

By combining carefully selected gamification elements with relevant learning content, the GIEB aims to create an engaging and effective learning environment. The design, grounded in self-determination theory, empowers students to take ownership of their learning, experience a sense of progress, and connect with their peers, ultimately fostering a deeper understanding of bullying behavior.

Table 2 summarizes the gamification elements applied in the GIEB, based on autonomy, competence, and relatedness.

**Table 2:** Gamification elements applied in the GIEB.

Deci and Ryan SDT theory	Gamification elements applied in the GIEB
Autonomy	**Free Choice of Role:** Students decide whether to experience the scenario as victim, bully, or bystander.
	**Control over Navigation:** Students can start, pause, repeat, or skip sections of the interactive e-book at will.
Competence	**Challenges and Questions:** Story-embedded questions and scenarios prompt critical thinking and application of knowledge.
	**Timely Feedback:** The system provides immediate feedback on student choices, confirming correct answers and explaining incorrect ones.
	**Points and Badges:** Students earn points for correct answers, which they can then redeem for badges, signifying progress and achievement.
Relatedness	**Social Sharing:** The GIEB allows students to share their earned points and badges with their peers, encouraging interaction and friendly competition.

## 4 Methods

### 4.1 Research design

This study employed a quasi-experimental pre-test/post-test design to examine the effects of gamified interactive e-books (GIEBs) on Chinese primary school students’ knowledge of and motivation toward addressing bullying behavior. Participants were randomly assigned to an experimental group and a control group. The experimental group engaged with the GIEB intervention, while the control group received traditional lectures on bullying. Pre-tests and post-tests were conducted for both groups to measure the targeted variables, with pre-test scores included as covariates in the analysis to account for any pre-existing differences between the groups.

This study also drew on findings from Beran and Shapiro (2005), who noted that students in their research demonstrated a high level of prior knowledge about bullying before the intervention, likely influenced by parental, school, or media attention to the topic. They also highlighted the possibility of students providing socially desirable responses during assessments. To address these issues, this study assessed participants’ baseline understanding and perceptions of bullying during the pre-test phase and used this information to refine the questionnaire design and overall research approach, ensuring the intervention more effectively targeted gaps in knowledge and motivation.

### 4.2 Participants

The sample for this study consisted of 60 third-grade students (typically aged 8–9 years old) from two classrooms in a public primary school in Hefei, Anhui Province, China, selected through convenience sampling. Informed consent was obtained from parents or legal guardians before participation, and students were informed of their right to withdraw at any time without penalty. Participants were randomly assigned to either the experimental group (*n* = 30), which engaged with the gamified interactive e-book (GIEB), or the control group (*n* = 30), which received traditional lecture-based instruction on bullying prevention.

As part of the pre-test, students’ baseline knowledge of bullying prevention was assessed to control for prior knowledge differences. However, other demographic variables, such as gender distribution and prior exposure to bullying-related education outside of school, were not collected in this study. While such factors may influence engagement with gamified systems (Manero et al., 2016; Zahedi et al., 2021), the primary aim of this research was to evaluate the overall effectiveness of gamified learning. Future studies could consider incorporating additional demographic factors to explore individual differences in learning outcomes.

To ensure adequate statistical power, a power analysis was conducted during the study design phase using G*Power software. The analysis was based on an analysis of covariance (ANCOVA) framework, with the statistical power set to 0.8 (80%), a significance level of 0.05, and an effect size of *f* = 0.4. According to Cohen (1988), *f* = 0.4 indicates a large effect size, suitable for studies where the intervention is expected to have a significant impact.

The choice of an effect size of *f* = 0.4 was based on the following considerations: First, Cohen’s (1988) classification of effect sizes has been widely applied in educational intervention research, particularly in contexts where interventions are anticipated to have substantial effects on learning motivation and performance. Second, existing studies support the effectiveness of gamified learning and digital educational tools, such as interactive e-books, in enhancing students’ academic performance. For instance, Subhash and Cudney's (2018) meta-analysis found that gamified learning interventions significantly improved students’ learning motivation and academic outcomes, with effect sizes ranging from medium to large (*f* = 0.25 to *f* = 0.4). Furthermore, studies by Zainuddin et al. (2020) and Bus et al. (2019) corroborate the positive impact of gamified learning on educational outcomes. Additionally, meta-analyses by Li et al. (2023) and Zeng and Sun (2023) reported overall effect sizes of *g* = 0.822 and *g* = 0.782, respectively, for gamified learning in enhancing students’ academic achievement and motivation, both of which fall within the range of large effect sizes.

Based on these parameters and supporting literature, the power analysis indicated that a minimum total sample size of 52 students (26 per group) was required to achieve sufficient statistical power. This study recruited a total of 60 students (30 per group), exceeding the minimum sample size requirement and ensuring that the statistical power was adequately maintained.

### 4.3 Research instruments

This study utilized two primary measurement instruments: the Student Bullying Knowledge Level Scale and the Motivation to Learn Scale. Both instruments employed a five-point Likert-type scale, with response options ranging from 1 (strongly disagree) to 5 (strongly agree), and included reverse-scored items.

#### 4.3.1 Motivation to learn scale

This scale was adapted from Tuan et al.’s (2005) Scientific Motivation to Learn Scale (SMTSL), which originally consisted of 35 items and demonstrated high internal consistency (Cronbach’s alpha = 0.89 for the overall scale and 0.70–0.89 for sub-dimensions). The adapted Motivation to Learn Scale used in this study also comprises 35 items. Example items include:

- Question 16: *“I believe it’s crucial to acquire knowledge about bullying, as it’s applicable to my everyday life.”*
- Question 34: *“I would like to learn about bullying because it is challenging.”*

The adapted scale demonstrated strong reliability in this study, with a Cronbach’s alpha of 0.922.

#### 4.3.2 Student bullying knowledge scale

This scale was adapted from Nguyen’s (2015) Bullying Behavior Awareness in Schools Questionnaire, which reported a Cronbach’s alpha of 0.859. The adapted Student Bullying Knowledge Scale used in this study consists of 30 items and achieved a Cronbach’s alpha of 0.963, indicating good internal consistency. Example items include:

- Question 1: *“I would perceive bullying if someone persistently speaks negatively about me.”*
- Question 23: *“I would inform an adult when a classmate experiences verbal abuse or physical assault.”*

### 4.4 Experimental procedure

**Figure 9:**
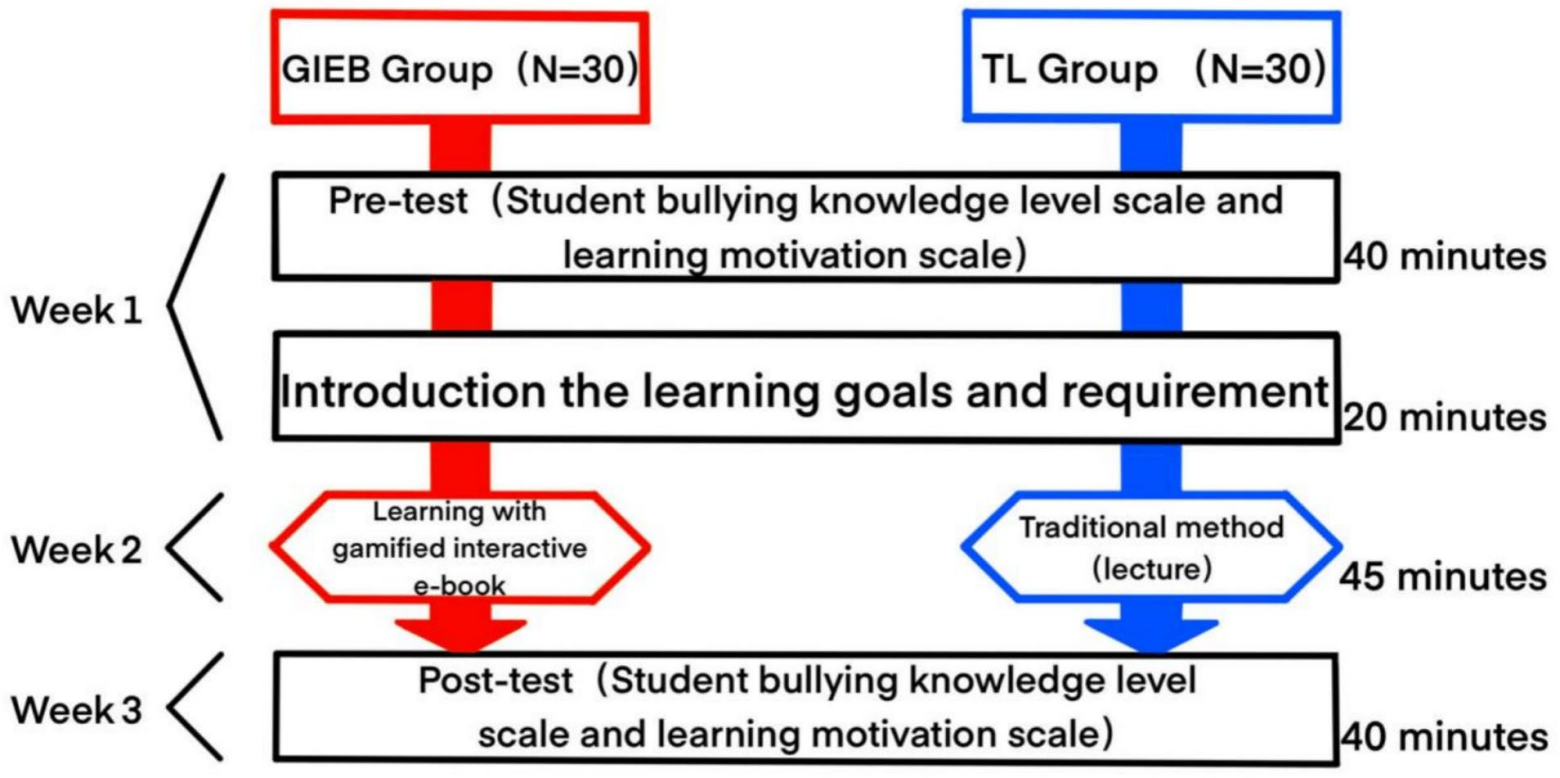
Experimental procedure.

The experiment spanned 3 weeks and involved distinct phases for both the GIEB (Gamified Interactive E-book) group and the TL (Traditional Lecture) group, as illustrated in Figure 9.

At the beginning of the experiment, all students completed a 40-min pre-test to assess their baseline understanding of bullying and their motivation to learn about the topic. This assessment included the Student Bullying Knowledge Level Scale and the Motivation to Learn Scale. Following the pre-test, the instructor conducted a 20-min session to introduce the learning goals and study requirements, ensuring that all participants understood the purpose and procedures of the subsequent learning activities.

During the second week, students engaged in learning activities based on their assigned groups. The GIEB group utilized the gamified interactive e-book for 45 min per session, mirroring the standard lesson duration in Chinese primary schools. Meanwhile, the TL group received traditional lectures on bullying for 45 min per session.

Finally, during the third week, all students completed a 40-min post-test to evaluate their knowledge of bullying and learning motivation following the interventions. This involved re-administering the Student Bullying Knowledge Level Scale and the Motivation to Learn Scale.

## 5 Data analysis and results

To determine the effectiveness of the GIEB intervention compared to traditional lectures, this study utilized analysis of covariance (ANCOVA). Compared to other data analysis methods, ANCOVA reduces error by controlling for covariates, thereby enhancing the accuracy of group comparisons. Additionally, ANCOVA improves the credibility of results by adjusting for initial group differences.

ANCOVA was chosen to examine the differences between the pre-test and post-test scores of the experimental and control groups, while statistically controlling for any pre-existing differences by including pre-test scores as covariates. This approach enhances the precision and sensitivity of the analysis, allowing for a more accurate assessment of the intervention’s impact.

Eta Squared (*η*^2^) was used to measure the effects of the GIEB and TL methods on students’ motivation to learn about bullying and their anti-bullying knowledge. Employing effect size helps eliminate the influence of sample size on measurement results, allowing for an understanding of the actual impact of the experimental findings rather than simply determining statistical significance.

Prior to conducting the ANCOVA, several assumptions were tested to ensure the validity of the results. These included:

1. Normality of data distribution
2. Homogeneity of variances
3. Meeting the criteria for the regression slope’s chi-square

Data analysis was performed using SPSS 26, and statistical significance was set at the 0.05 level. To further enhance the reliability of the findings, an independent sample t-test was conducted on the pre-test scores of both groups to confirm that there were no significant initial differences between them.

### 5.1 Effects of GIEB on students’ motivation to learn about bullying

To analyze the impact of the GIEB on students’ learning motivation, several statistical tests were conducted.

First, the normality of the pre-test and post-test scores for learning motivation in both the control and experimental groups was assessed using the Shapiro–Wilk test. The results (*p* = 0.701, *p* = 0.976, *p* = 0.535, *p* = 0.054) indicated that the data for both groups and both time points met the assumption of normality, as all *p*-values were greater than 0.05.

Next, Levene’s test was employed to examine the homogeneity of variances for the learning motivation pre-test and post-test scores. The results (*p* = 0.451 and *p* = 0.887, respectively) confirmed that the data met the assumption of equal variances, as both *p*-values exceeded 0.05. Additionally, the regression slope chi-square test (*F* = 0.006, *p* = 0.938 > 0.05) indicated that the data met the required assumptions for ANCOVA.

An independent sample t-test was then conducted on the pre-test scores of learning motivation for both groups to assess for any significant initial differences. The results (*p* = 0.964 > 0.05) indicated no significant difference in learning motivation between the GIEB and TL groups at baseline.

Finally, an analysis of covariance (ANCOVA) was performed to examine the effect of the GIEB intervention on post-test learning motivation scores, while controlling for pre-test scores. As shown in Table 3, the between-subjects effect test revealed that pre-test scores did not have a significant effect on post-test learning motivation (*F* = 0.075, *p* = 0.785 > 0.05). However, the ANCOVA results demonstrated a significant difference in adjusted mean post-test scores between the two groups. The GIEB group had a significantly higher mean score (24.103) compared to the TL group (20.753), *F* = 63.530, *p* < 0.001, *η*^2^ = 0.527. This suggests that the GIEB intervention effectively enhanced students’ motivation to learn about bullying compared to traditional lectures.

**Table 3:** Descriptive data and ANCOVE results for the learning motivation posttest.

Variable	Group	*N*	Mean	S.D	Adjusted mean	*F*	*p*	*η* ^2^
Post-test	GIEB	30	24.1036	1.56	24.103	63.530	<0.001	0.527
	TL	30	20.7526	1.67	20.753			

### 5.2 Effects of GIEB on students’ anti-bullying knowledge

The impact of the GIEB on students’ anti-bullying knowledge was evaluated using a series of statistical tests.

First, the normality of pre-test and post-test scores for bullying knowledge in both the control and experimental groups was assessed using the Shapiro–Wilk test. The results (*p* = 0.115, *p* = 0.155, *p* = 0.241, *p* = 0.661) confirmed that the data met the normality assumption, as all *p*-values were greater than 0.05.

Levene’s test was then conducted to examine the homogeneity of variances for the pre-test and post-test scores of bullying knowledge. The results (*p* = 0.484, *p* = 0.439) showed that the data satisfied the assumption of equal variances, as both *p*-values exceeded 0.05. Additionally, a regression slope chi-square test (*F* = 0.4, *p* = 0.53 > 0.05) revealed that the data met the assumptions required for ANCOVA.

An independent sample t-test was performed on the pre-test scores for bullying knowledge to confirm that there were no significant initial differences between the groups. The results (*p* = 0.065 > 0.05) indicated no significant difference in bullying knowledge between the GIEB and TL groups at baseline.

Finally, an analysis of covariance (ANCOVA) was conducted to examine the effect of the GIEB intervention on post-test bullying knowledge scores, while controlling for pre-test scores. Table 4 displays the descriptive data and ANCOVA results. The between-subjects effect test revealed that students’ pre-test scores on bullying knowledge did not significantly affect post-test scores (*F* = 0.204, *p* = 0.654 > 0.05). However, the ANCOVA results showed a significant difference in adjusted mean post-test scores between the two groups. The GIEB group (15.766) demonstrated significantly higher bullying knowledge than the TL group (13.689), *F* = 30.182, *p* < 0.001, *η*^2^ = 0.376.

**Table 4:** Descriptive data and ANCOVE results for the bullying knowledge posttest.

Variable	Group	*N*	Mean	S.D	Adjusted mean	*F*	*p*	*η* ^2^
Post-test	GIEB	30	15.7902	1.23	15.766	30.182	<0.001	0.376
	TL	30	13.6639	1.54	13.689			

These findings indicate that the GIEB intervention was effective in enhancing students’ understanding of bullying compared to traditional lectures.

## 6 Discussion

This study aimed to evaluate the effectiveness of a gamified interactive e-book (GIEB) in enhancing Chinese primary school students’ motivation and awareness of bullying prevention behaviors. The findings demonstrate that the GIEB intervention significantly improved both learning motivation and students’ awareness of anti-bullying behaviors compared to traditional lecture-based instruction. The results align with Saibon et al. (2017), who found that employing creative and engaging teaching methods enhances student motivation, which subsequently heightens their awareness of bullying behaviors.

Our research findings demonstrate a significant improvement in students’ awareness of bullying prevention among those in the GIEB group compared to their pre-test results. This result is consistent with the findings of Donohoe and O'Sullivan (2015) and Rončević Zubković et al. (2022), supporting the idea that features such as role-playing, scenario-based decision-making, feedback and rewards, and social interaction embedded in the GIEB effectively enhance students’ motivation to engage in bullying prevention behaviors. This motivation stimulates students’ enthusiasm for learning, encouraging them to participate more actively in learning activities, thereby improving their behavioral awareness related to bullying prevention.

The significantly higher learning motivation observed in the GIEB group aligns with previous research highlighting the positive impact of gamification on student engagement and motivation (Takbiri et al., 2023). The GIEB’s design, rooted in the Self-Determination Theory (SDT) framework, deliberately incorporated elements to foster students’ autonomy, competence, and relatedness. Providing students with choice in role selection, navigation control, and learning pace supported their sense of autonomy. The integration of challenges, quizzes, feedback mechanisms, and reward systems (points and badges) contributed to feelings of competence and progress. Enabling social sharing of achievements fostered a sense of relatedness among students. As highlighted by Teba (2023), meeting these psychological needs through gamification is crucial for promoting intrinsic motivation and a willingness to learn.

The specific gamification elements included in this study—badges, points, feedback, challenges, and immersive story scenarios—align with Chen et al.’s (2023) assertion that effective gamification must address the three psychological needs outlined in SDT and be closely integrated with the learning content. The GIEB’s success in motivating students suggests that its design effectively achieved this alignment.

The study’s findings also reveal that students in the GIEB group significantly outperformed their peers in the TL group on measures of bullying prevention awareness. This outcome supports the hypothesis that increasing students’ motivation to engage with bullying prevention content would lead to improved awareness of bullying behaviors and appropriate responses. Saibon et al. (2017) similarly demonstrated a positive correlation between motivation and behavioral awareness in the context of bullying prevention education.

Beyond enhanced motivation, the GIEB’s unique features likely contributed to students’ behavioral awareness. The opportunity for students to experience bullying scenarios from multiple perspectives through role-playing, as well as engage in discussions and share insights with their peers, provided a deeper and more nuanced understanding of the complexities of bullying behavior. Donohoe and O'Sullivan (2015) emphasized the value of role-playing and discussions in facilitating reflection on bullying experiences and enhancing students’ ability to recognize and respond to bullying behaviors. The GlEB’s combination of interactive storytelling, gamified elements, and social features created a dynamic learning environment that fostered both engagement and behavioral awareness.

Bullying in China is shaped by unique cultural factors, including a strong emphasis on academic achievement, collectivist values, and hierarchical relationships between teachers and students (Bergeron and Schneider, 2005; Wang, 2019). The GIEB’s design was mindful of these cultural influences. For example, the choice of role-playing scenarios was carefully considered to reflect bullying situations common in Chinese schools, and the story content incorporated themes of respect for authority and group harmony. The use of points and badges as rewards aligned with the emphasis on achievement, while the social sharing feature promoted collaboration and a sense of collective progress. Future research could explore how cultural adaptations of GIEBs might further enhance their effectiveness in different cultural contexts.

## 7 Limitation and recommendation

While this study provides valuable insights into the potential of GIEBs for bullying prevention education, several limitations should be acknowledged.

First, the study involved a relatively small sample of 60 third-grade students from a single public primary school in Hefei City, Anhui Province, China. This limited sample size and specific context restrict the generalizability of the findings to a broader population of Chinese primary school students. Future research should aim to replicate this study with larger and more diverse samples, including students from different regions, school types, and socioeconomic backgrounds, to improve the external validity of the results.

Second, this study did not systematically collect demographic information such as gender distribution and prior exposure to bullying-related education. While prior knowledge of bullying was assessed through pre-tests, a more comprehensive analysis incorporating demographic variables could provide deeper insights into how individual differences influence engagement with gamified learning. Future research should consider incorporating these demographic factors, as prior studies suggest that variables such as gender and past experiences can influence students’ responses to gamified systems (Manero et al., 2016; Zahedi et al., 2021).

One limitation of this study is the inability to directly measure the impact of gamification-supported education on actual bullying behavior, as the study primarily focused on students’ acquisition of anti-bullying knowledge. This limitation arises from cultural and contextual factors in China (Bergeron and Schneider, 2005; Wang, 2019), where some schools expressed concerns about implementing bullying behavior monitoring initiatives due to potential reputational and legal implications (Shariff, 2009; Ministry of Education of the People's Republic of China, 2017). As a result, collecting relevant behavioral data proved challenging. Future research should prioritize investigating the behavioral effects of gamification-supported education and employ diverse measurement approaches, such as behavioral observations, teacher assessments, and self-reports, to comprehensively evaluate changes in bullying behavior. Moreover, fostering stronger collaboration with schools will be essential. Establishing transparent communication with school administrators to highlight the educational significance of the study and ensuring strict data confidentiality may help alleviate institutional concerns, thereby facilitating data collection and enabling a more thorough assessment of gamification’s role in bullying prevention.

The intervention period was relatively short, spanning only 3 weeks. While significant changes in knowledge and motivation were observed within this timeframe, a more extended intervention period could potentially lead to more robust and sustained effects. Future studies should consider implementing GIEB interventions over a longer duration to assess the longevity of its impact.

This study focused on a limited selection of gamification features, primarily points, badges, and role-playing. Expanding the range of gamification elements, such as incorporating leaderboards, customizable avatars, narrative branching, and personalized feedback mechanisms, could further enhance student engagement and personalize the learning experience.

The current GIEB design assumes that all students respond similarly to gamification elements. However, individual learning preferences and motivational factors can vary widely. Future research could explore the implementation of adaptive gamification design, which tailors the selection and application of game mechanics based on individual student characteristics and learning needs. This personalized approach could potentially lead to even greater improvements in motivation and knowledge acquisition.

While this study demonstrated immediate changes in knowledge and motivation, the long-term impact of the GIEB intervention on students’ attitudes, behaviors, and bystander intervention skills warrants further investigation. Longitudinal studies are needed to assess whether the observed effects are sustained over time and translate into a reduction in bullying incidents and an increase in prosocial behaviors. Qualitative data, gathered through student interviews or focus groups, could offer valuable insights into how the GIEB experience shaped students’ understanding of bullying and their willingness to intervene when witnessing such behaviors.

## 8 Conclusion

This study examined the effects of gamified interactive e-books (GIEBs) on Chinese primary school students’ knowledge and motivation regarding bullying. The findings provide compelling evidence that GIEBs can significantly enhance both students’ understanding of bullying and their willingness to engage in learning about this important social issue.

The study’s results hold significant implications for educational practice, particularly in the realm of bullying prevention. GIEBs offer a promising approach to engaging young learners in a topic that can often be challenging to address through traditional methods. By integrating interactive storytelling, game mechanics, and opportunities for social interaction, GIEBs create a dynamic learning environment that fosters both comprehension and motivation. Educators and curriculum developers can leverage these findings to design and implement engaging digital resources that effectively address bullying prevention in primary school settings.

The GIEB’s design was explicitly grounded in the Self-Determination Theory (SDT) framework (Deci and Ryan, 2012), incorporating elements to support student autonomy, competence, and relatedness. The study’s positive results provide further empirical support for SDT’s application in educational technology design. By strategically incorporating features that address these fundamental psychological needs, GIEBs can effectively foster intrinsic motivation, leading to enhanced learning outcomes. This study contributes to a growing body of evidence demonstrating the efficacy of integrating SDT principles into gamified learning interventions.

In conclusion, this study offers compelling evidence for the effectiveness of GIEBs in enhancing anti-bullying knowledge and motivation among Chinese primary school students. The findings have practical implications for educational practice and contribute to our theoretical understanding of how gamified interventions can leverage SDT principles to promote meaningful learning.

## Data Availability

The raw data supporting the conclusions of this article will be made available by the authors without undue reservation.
